# A systematic review and meta-analysis of the relationship between genes and reflexive attention

**DOI:** 10.3389/fnins.2025.1449354

**Published:** 2025-05-30

**Authors:** Spencer Myres, Katherine E. Christensen, Rebecca A. Lundwall

**Affiliations:** ^1^Baylor College of Medicine, Houston, TX, United States; ^2^Pacific Graduate School of Psychology-Stanford PsyD Consortium, Palo Alto, CA, United States; ^3^Psychology Department, Brigham Young University, Provo, UT, United States

**Keywords:** meta-analysis, genetic influence, reflexive attention, development, children and adults, neurotransmitters

## Abstract

Defined as the semi-autonomous orientation to either a moving or rapidly appearing stimulus, reflexive attention (RA) is a crucial process for humans. While there are multiple outcomes used to assess RA, their relationships have not been tested. Disruptions in RA often relate to neurodevelopmental disorders, such as attention-deficit/hyperactivity disorder or autism spectrum disorder, so RA might be moderated by age. Additionally, the current academic consensus is that multiple genes influence RA, but this has not been assessed in a meta-analysis. A better understanding of RA using previously collected data will allow us to improve the design of future research studies.

**Methods:**

In accordance with PRISMA, we conducted a broad search for potentially relevant articles pertaining to genes associated with RA. Selected studies included those (1) published in English, (2) involving human participants, and (3) referencing specific genetic markers in association with a measure of RA. For subgroup comparisons, we analyzed 14 studies assessing children and 23 assessing adults. We also compared 18 dopamine-related to 19 non-dopamine related studies.

**Results:**

The main analysis produced a non-significant overall effect size; however, our most interesting finding was that results varied by age group. We explore this as well as difference by outcome type and the relation of the gene studied to dopamine.

**Conclusion:**

Our findings vary by age group. However, due to heterogeneity we recommend more studies to answer some questions about a broader range of neurotransmitters, to include younger age groups, and to clarify difference by outcome type. We discuss issues of relevance to researchers to guide future meta-analyses.

**Systematic review registration:**

Prospero: International prospective register of systematic reviews. Available from: https://www.crd.york.ac.uk/PROSPERO/view/CRD42018090220.

## Introduction

Reflexive attention^1^ (RA) is a process that has substantial implications for the cognitive, social, and emotional well-being of humans (Bagherzadeh-Azbari et al., 2023; Lundwall and Rasmussen, 2016; Pozuelos et al., 2014). It allows individuals to attend to potential dangers, obtain food within their environments, and even successfully interact and have relationships with others (Bagherzadeh-Azbari et al., 2023; Feng and Zhang, 2014; Phelps et al., 2006). Research suggests that children can demonstrate relative differences in RA development early on in life (Dannemiller, 2004; Holmboe et al., 2010; Jones and Kiln, 2013), variations of which impact neurodevelopmental disorders, such as attention-deficit/hyperactivity disorder (ADHD) or autism spectrum disorder (ASD).

In contrast to sustained attention (i.e., when an individual effortfully focuses on a stimulus for an extended period of time), RA occurs automatically as an individual orients their focus to the sudden appearance or movement of a stimulus (Richards and Hunter, 2002; Sechenov, 1863/1965; Smith and Chatterjee, 2008). Thus far, several genetic association studies have been completed with the aim to better understand the biological underpinnings of RA (Beane and Marrocco, 2004; Bobb et al., 2005; Lundwall et al., 2012; McCauley et al., 2004). These studies found, for example, that genetic markers associated with acetylcholine, dopamine, norepinephrine, and serotonin are associated with RA. However, reviews of these genetic studies, including meta-analyses, are much rarer.

Given that disorders involving attentional difficulties have been associated with a variety of genes and neurotransmitters (Bobb et al., 2005; Lundwall et al., 2017; McCauley et al., 2004) and that the variety of evidence has not previously been combined in a meta-analysis, we judged that the pooling of data may allow for a clearer estimate of neurotransmitter influence on RA.

Our study screened genetic studies of RA using strict criteria for inclusion in our meta-analysis. In addition to determining genetic influence by outcome type, we associate the genes with neurotransmitters to compare dopamine-related to non-dopamine-related effects. Similarly, our interest in development from infancy to adulthood drove planned group analyses that would compare child to adult age groups.

## Reflexive attention tasks

### AUD Cz, target amplitude

Even-related potential (ERP) studies provide a reliable and sensitive method to investigate cognitive processes such as novelty detection, attentional allocation, and target discrimination (Gallinat et al., 2003). In the auditory modality, a number of ERP components (e.g., N1, P1) have been associated with the different stages of novelty processing. A common feature of these ERP components is that they are elicited even when sound is irrelevant for the subject’s task (Birkas et al., 2006). Readings at the central electrode (Cz) in an auditory ERP study refer to the auditory target amplitude. Novelty processing reveals stages of sensory detection and attention reorientation (Birkas et al., 2006). Differences in ERP amplitudes generally offer insights into genotype-dependent variations in cognitive processing, although not necessarily into early attentional processing (Anokhin et al., 2001; Tye et al., 2011). This suggestions the possible heritable nature of reflexive attention (Gallinat et al., 2003; Reinvang et al., 2005). These measures are crucial for understanding the neural dynamics of sensory and attentional systems.

### Benefit

The cued-orienting task (Posner, 1980) involves precues and targets flashing briefly on a computer display. It is taken as evidence that attention was captured by a precue if subjects were faster at responding to a subsequent target even though the stimuli presentation is too brief to depend on eye movement. Depending on the specific variation of the task, the precues can be valid (indicate where the target will subsequently appear), invalid (indicate the contralateral side from where the target ultimately appears), or neutral (does not provide location information). See Bellgrove et al. (2009) for a variation on this task. Benefits represent the tendency for participants to respond with shorter latencies (response times; RTs) when responding to validly cued targets than to targets following neutral precues. Benefits are calculated as neutral RT–valid RT.

### Cost

**Figure 1:**
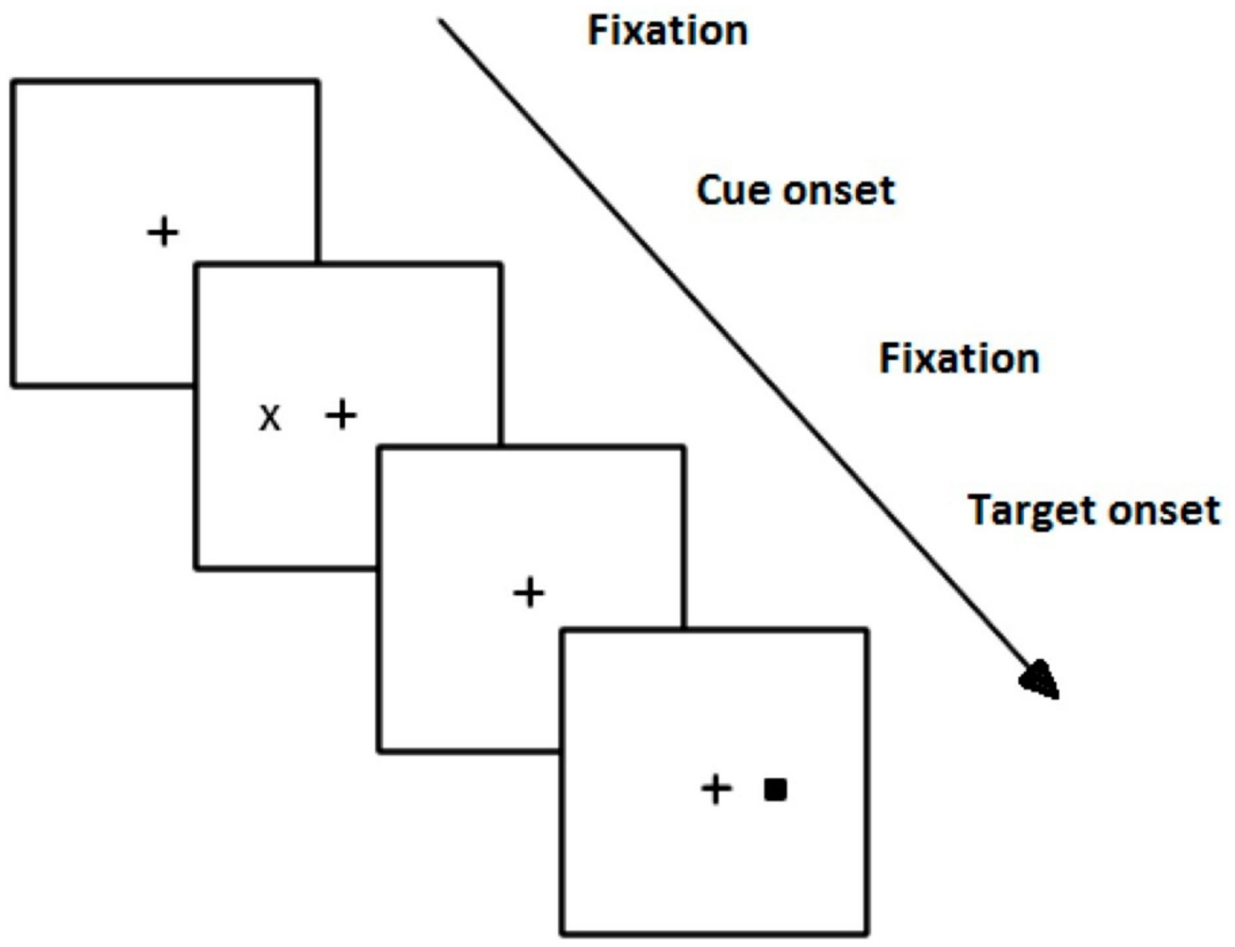
Illustration of a Posner-like paradigm task. This diagram illustrates the presentation of an invalidly cued target. If the trial had been valid, the cue (“X”) and target “■” would have appeared ipsilaterally. A neutral cue would have had two bilateral cues preceding the target.

A cost occurs when the participant is slower at responding to a target following an invalid cue than they are, on average, to a neutral cue. Costs usually follow invalid cues that suggest the target will appear on the side opposite where it subsequently appears. It is calculated as invalid RT − neutral RT. See Figure 1 for an illustration.

### Flanker

The flanker task, originally introduced by Eriksen and Eriksen (1974), is a cognitive paradigm used to study visual attention and interference processing. In the version used most often in the studies included in the meta-analysis (Eriksen and Schultz, 1979), participants are required to respond to a central target (e.g., a letter or arrow) that has flanking distractors on either side. These distractors can be congruent (pointing in the same direction as the target; e.g., >>>> > or <<<<<) or incongruent (pointing in the opposite direction of the target; e.g., <<> < <). The congruency influences RT and accuracy, with faster and more accurate responses observed in congruent trials compared to incongruent ones. The task effectively measures the ability to suppress irrelevant information since incongruent distractors interfere with target processing, slowing RT and increasing error rates. Variations of the flanker task include noise conditions, neutral distractors, and blocked trial designs, further exploring the dynamics of attentional allocation and response competition. Scores represent the difference between response times when all flanking cues point in the opposite direction of a central cue versus when all cues point in the same direction. Thus, it is typically calculated as incongruent RT–congruent RT.

### Gap effect

In the gap-no-gap task, there are two conditions that each involve shifting attention from a central stimulus to a peripheral target (Chernenok et al., 2019; Owen, 2015; Ross-Sheehy et al., 2015). The “gap” condition involves a central fixation stimulus that disappears before a peripheral target appears, creating a temporal gap. In the “overlap” condition, the fixation stimulus remains visible alongside the peripheral target, causing visual competition. The “gap effect” refers to the increased latency (via saccades) observed in the overlap condition compared to the gap condition, reflecting the recruitment of cortical and subcortical processes required for disengaging attention from the central stimulus. The gap–overlap task is a cognitive paradigm used to study attention-shifting mechanisms. The gap is thought to aid the disengagement of attention relative to a trial without a gap (Ross-Sheehy et al., 2015). The RT score is calculated as overlap RT–gap RT.

### Valid orienting

One way to calculate scores is simply to time the latency of a look toward a valid cue. This is different from a Posner paradigm benefit because the latter subtracts RT to neutral cues. Thus, the valid orienting outcome is not adjusted for an individual’s base RT in the same way that a benefit is.

### Validity effect

Sometimes calculated when using a Posner paradigm, the validity effect is the difference in RT between validly and invalidly cued trials. Thus, it is calculated as invalid RT–valid RT. The task is usually a peripherally cued variation of the Posner paradigm, but benefits and costs are not necessarily provided (Schneider et al., 2015).

### Neurotransmitter influence on reflexive attention

Some researchers have suggested that differences in attentional development are influenced by multiple genes (Beane and Marrocco, 2004; Bobb et al., 2005; McCauley et al., 2004; Saez et al., 2014), implying that individuals with attentional difficulties may carry alleles that affect their performance on attention-related tasks. If this is the case, neurotransmitters may have significant bearing on RA since they often prove the relevant pathway for the influence of genes on behavior (Beane and Marrocco, 2004; Saez et al., 2014).

Research has linked the influences of specific genes and neurotransmitters to the development of RA among infants and children; however, the findings are often mixed. For example, several researchers discuss genes, including *CHRNA4, COMT, MAOA,* and *SLC6A3* (Bellgrove et al., 2007; Markant et al., 2014; Quan et al., 2017; Zhang et al., 2011), which are associated with cholinergic, dopaminergic, noradrenergic, and serotonergic (and, indirectly, with glutaminergic) neurotransmitters. In addition, Bellgrove et al. (2007) found that two variable number tandem repeats (VNTRs) were associated with the *SLC6A3* gene among typically developing children performing an RA task while Markant et al. (2014) showed that variations in the *COMT* gene, which influences dopamine in the brain, may contribute to individual differences in reflexive orienting during childhood.

These findings suggest that genes and neurotransmitters influence the development of RA in infancy and early childhood; however, no study to date has comprehensively assessed the relative importance of candidate genes, represented in various neurotransmitters, have on RA. Thus, a systematic review and meta-analysis is a key step to understanding the implications of genetic influence on difficulties with RA.

### Reflexive attention across age groups

Research suggests that children can demonstrate deficits in RA development early on in life (Dannemiller, 2004; Holmboe et al., 2010; Jones and Kiln, 2013). These differences may sometimes involve the development of various neurodevelopmental disorders, such as ADHD or ASD. The development of RA appears within the first year of life (Harman et al., 1994; Quan et al., 2017; Ross-Sheehy et al., 2015; Rothbart et al., 2011) and is likely influenced by a variety of genes (Bellgrove et al., 2007; Markant et al., 2014; Zhang et al., 2011).

Several researchers indicate that the RA characteristics of children of six years and older do not differ much from those of adults (Brodeur and Enns, 1997; Plude et al., 1994; Waszak et al., 2010). Most development of RA may occur in infancy when looking durations tend to decrease with age (Colaizzi et al., 2014; Courage et al., 2006) and covert spatial orienting develops (Richards, 2005). Some evidence suggests that there are changes in RA such that younger children or more likely to experience costs following valid cues (Lundwall et al., 2018; although see Enns, 1990 and Waszak et al., 2010 for alternate findings). In addition, children become faster at locating a peripheral target in a field of distractors between 7 and 11 years old (Dye and Bavelier, 2010). However, there appear to be minimal differences in peripheral cueing between 7 and 73 years old (Brodeur and Enns, 1997).

## Methods

Our aim for this meta-analysis was to include all candidate gene association studies with humans that test any specific genetic markers for association with RA. We based this on advice from several sources to ensure quality and accuracy of data (Boccia et al., 2010; Committee on Standards for Systematic Reviews of Comparative Effectiveness Research, 2011; Gwinn et al., 2014; Minelli et al., 2009; Thompson et al., 2011) and conducted it in accordance with the Preferred Reporting Items for Systematic Reviews and Meta-Analyses (PRISMA) framework. PRISMA consists of both evidence-based guidelines for and transparency of reporting in meta-analyses. This study was pre-registered on 5 December 2017, at Prospero: International prospective register of systematic reviews. Available from: https://www.crd.york.ac.uk/PROSPERO/view/CRD42018090220 and modified on 12 March 2018.

### Search strategy

First, we surveyed core attention literature discussing the terminology for different tasks measuring RA. We also documented the years covered and how searches functioned within a given database (e.g., if we could use phrases in quotation marks in combination with a Boolean/Lucene search and if we could use parentheses for nesting terms).

Pilot testing on inclusion procedures occurred during Fall and Winter 2017–2018. To avoid any selection bias (Boccia et al., 2010; Eicher and Gruen, 2015), we conducted a broad, comprehensive literature search for potentially relevant articles pertaining to genes and RA. Our strategy consisted of searching each of the databases to determine the best search terms to yield the greatest number of relevant studies (see selection criteria below). However, we eventually searched simultaneously those databases that had the same search functionality (e.g., Boolean terms, nesting).

Eventually, we found that the following databases adequately identified relevant literature while avoiding unnecessary duplicate hits: Academic OneFile (Gale); APA PsycInfo; Academic Search Ultimate; Biomedical Reference Collection: Basic; CINAHL; ERIC; MEDLINE; Psychology and Behavioral Sciences Collection; Elsevier ScienceDirect; Elsevier Scopus; Embase (Medline hits removed); ProQuest (Dissertations & Theses); PubMed; Web of Science; and CDC-Authored Genomics and Precision Health Publications Database. Searches were not restricted by date of publication. We used the following search terms: genetic AND [(“orienting” AND “attention”) OR “reflexive attention” OR “exogenous attention” OR “peripheral attention” OR “selective attention”]. We examined each title and abstract and, if the article was apparently relevant, the full text with the assumption of inclusion until inclusion criteria were violated (see subsection “Search Strategy”). We also searched preprint repositories to avoid contributing to publication bias (see section “Publication Bias”). In bioRxiv, which also searches medRxiv and PsyArXiv repositories, we altered our search strategy because (a) nesting was not supported, (b) the same Boolean operator had to be used between each search term, and (c) Boolean terms could not be used with phrase search. Instead, we searched for titles and abstracts in these databases using two terms at a time. No additional useful hits were obtained from these repositories when searching the full texts.

**Figure 2:**
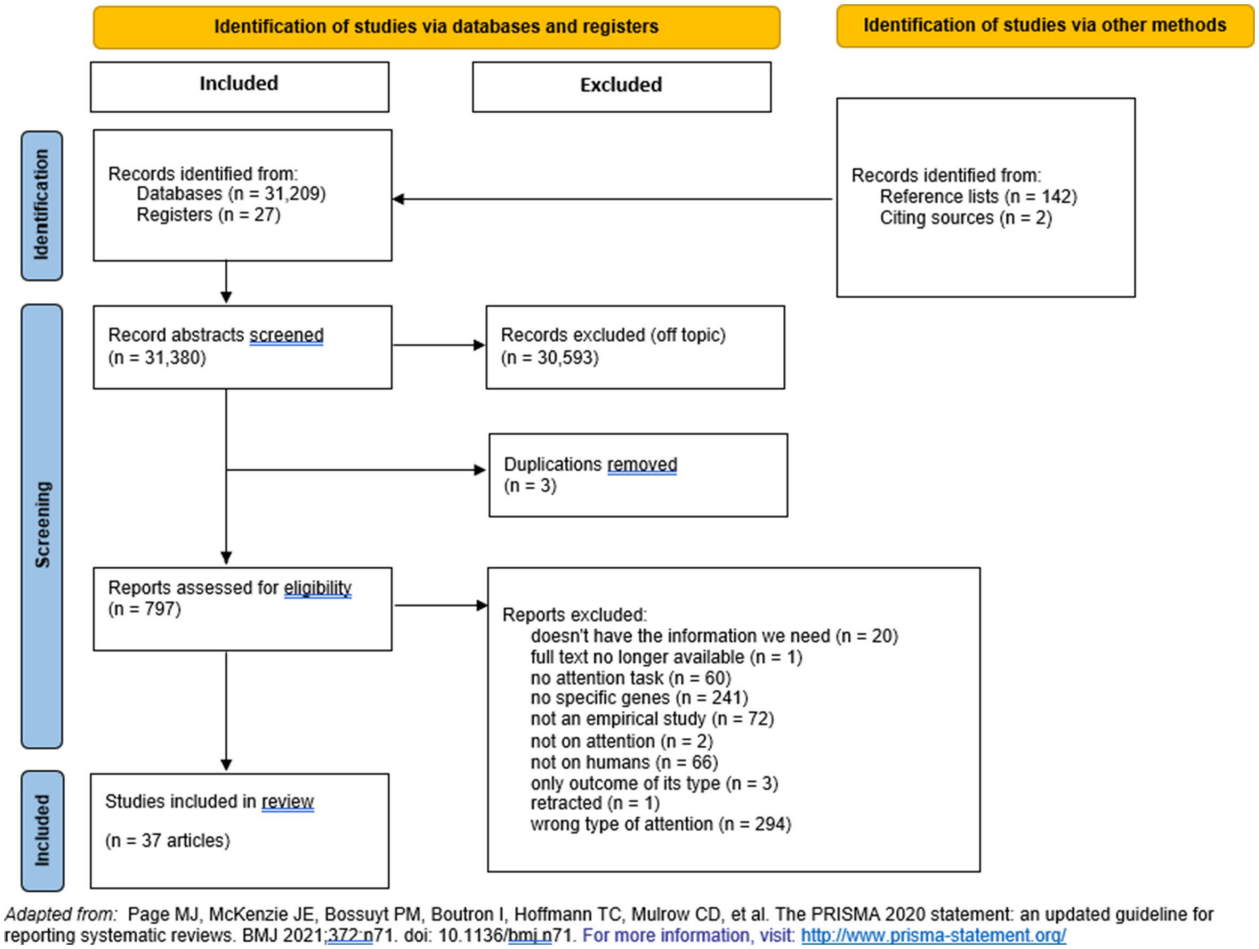
Flowchart illustrates the number of studies evaluated in this meta-analysis.

We updated our search in April 2024, prior to article submission. In all searches, at least two team members confirmed exclusion of a study. For included articles, we also searched reference lists until saturation. For an illustration of our selection process, see Figure 2.

### Selection criteria

We included studies if they were published in English^2^, involved human participants, and referenced specific genetic markers in association with a measure of RA. Candidate gene association studies and genetic disorder association studies were included if they referenced RA performance between genetic groups or conditions. While genome-wide associations are sometimes preferred (Mammen and Arcoleo, 2019), we could not include these studies since they only reported significant genes and could lead to issues with selective reporting.

Article reviewers worked independently to screen the titles and abstracts of all articles discovered using our search strategy. Studies included by either of the two reviewers were retrieved for full-text screening. Independent reviewers screened the full-text version of articles. If they could not reach consensus in determining whether the article was relevant to the current analysis, a third reviewer resolved the discrepancy. See Table 1 for exclusion data.

**Table 1:** Full-text articles evaluated but excluded from the meta-analysis.

Reason	*N*
Did not have the information we need	20
Full text no longer available	1
No attention task	60
No specific genetic marker	241
Not an empirical study	72
Not on attention	2
Not on humans	66
Only outcome of its type	3
Retracted	1
Wrong type of attention	294

### Data extraction

Before data extraction, we developed a standardized form to enter study characteristics, including group sample sizes, type of RA measure, means and standard deviation, direction of effect, and specific genetic markers in the analyses. See Table 2 for a list of articles included.

**Table 2:** Articles included in the meta-analysis.

Study name	Overall *N*	Mean age (SD) range	Ethnicities	Genetic marker
Anguera et al. (2016)	111	10.4 (2.3) yrs	NR	16p11.2 DS
Bellgrove et al. (2007)	51	13.7 (2.1) 9–16 yrs	NR	SLC6A3
Bellgrove et al. (2009)	115	13.5 (1.9) yrs	NR	SLC6A3
Birkas et al. (2006)	57	6–7 yrs^3^	NR	DRD4
Bish et al. (2007)	30	9.5 (2.2) 7–14 yrs	NR	22q11.2
Chernenok et al. (2019)	58	39.6 (19.4) mos	NR	FMR1
Cornish et al. (2008)	17	27 (NR) 3–55 mos	NR	FMR1
Couette et al. (2008)	28	46.5 (NR) 34–59 mos	NR	HTT
Espeseth et al. (2007)	42	62.2 (8.4) 48–75 yrs	NR	CHRNA4
Fan et al. (2003)	183	27.2 (5.7) yrs	NR	DRD4
Gallinat et al. (2003)	219	39.4 (8.3) yrs	100% Caucasian	COMT
Gallinat et al. (2007)	281	40.8 (0.19) yrs	NR	GRIN3A (aka NR3A)
Gao et al. (2018)	83	69.9 (0.29) 50–80 yrs	100% Asian	APOE
Greenwood et al. (2005)	177	59.4 (0.29) 41–85 yrs	NR	APOE
Lundwall et al. (2012)	161	25.32 (NR) 18–61 yrs	20% Asian; 5% Black; 61% White (15% Hispanic)	COMT
Lundwall et al. (2017)	201	13.0 (1.7) 9–16 yrs	100% Caucasian	COMT
Mannarelli et al. (2018)	20	26.0 (1.8) yrs	NR	22q11.2
Marco-Pallarés et al. (2010)	45	22.2 (NR) 18–35 yrs	NR	COMT
Markant et al. (2014)	88	7 (NR) 6.7–7.26 mos	6% Asian; 1% Black; 93% White	COMT
Maurage et al. (2017)	152	49.4 (3.7) yrs	NR	HTT
Newman et al. (2014)	518	21.6 (0.1) yrs	100% Caucasian	SLC6A3
O’Donoghue et al. (2014)	97	41.2 (12.0) 18–60 yrs	100% Caucasian	ZNF804A
Ortega-Mora et al. (2021)	127	23 (0.8), 20–30 yrs	NR	CNR1
Owen (2015)	124	18.0 (NR) 3.2–67.1 mos	NR	FMR1
Parasuraman et al. (2005)	89	35.2 (2.5) yrs	NR	CHRNA4
Quan et al. (2017)	276	183.4 (4.9) 174–194 days	100% Asian	COMT
Quintero et al. (2014)	129	10.5 (2.3) 7–15 yrs	NR	22q11.2
Reuter et al. (2007)	100	22.6 (4.7) yrs	100% Caucasian	COMT
Rose et al. (2019)	62	7.3 (3.1) 2–12 yrs	NR	MECP2
Schneider et al. (2015)	157	21 yrs. (median)	NR	CHRNA4
Schulz et al. (2012)	324	23.9 (2.7) 17–31 yrs	100% White	GRIN2B
Simon et al. (2005)	27	10 (2.7) 7–15.6 yrs	NR	22q11.2
Sobin et al. (2004)	69	7.9 (1.8) 5–14.1 yrs	NR	22q11.2
Thimm et al. (2010)	80	23.2 (2.9) 18–55 yrs	100% White	DTNBP1
Thimm et al. (2011)	46	23.3 (2.8) 18–55 yrs	NR	CACNA1C
Tsai et al. (1995)	65	[No mean provided]	NR	HTT
Zareyan et al. (2021)	156	24.2 (3.0) 20–35 yrs	26% Asian; 74% White	COMT

NR, not reported.

### Genetic markers

After selecting the studies for final inclusion in the meta-analysis, we employed the following rules to help avoid problems with the non-independence of samples. We also checked for the use of duplicate samples across multiple papers since this would result in non-independence of data. However, none of the studies in our sample for meta-analysis included words or phrases that led us to believe the same data had been published more than once.

- If a single study had two outcomes for the same marker, we selected the more common of the two outcomes for inclusion to ensure at least two studies per outcome type.
- If two or more genetic markers were tested in the same sample, we selected the non-dopamine related genetic marker. If there were multiple non-dopamine markers, then we selected the most common marker. If two single nucleotide polymorphisms (SNPs) from the same genetic marker were in the study, we included the more commonly studied SNP.

In our final dataset, we included the following genetic markers: *16p11.2*, *22q11.2*, *APOE, CACNA1C, CHRNA4, CNR1, COMT, DRD4, DTNBP1, FMR1, GRIN2B, GRIN3A, HTT, MECP2, SLC6A3,* and *ZNF804A.* These are related to the neurotransmitters acetylcholine dopamine, epinephrine, norepinephrine, GABA, glutamate, and serotonin and to neurotransmitter modulation generally. Due to the greater number of studies involving dopamine generally, we classified the studies in the meta-analysis into dopamine-related and non-dopamine related studies. We judged that finer tuned analysis was unwise given our sample size. Here we describe the biological mechanisms by which these genetic markers are proposed to have an influence on RA.

#### 16p11.2

Deletions in this region of chromosome 16 are associated with a pattern of developmental delays, learning disabilities, and intellectual and attention-related conditions such as ASD (Anguera et al., 2016; Hanson et al., 2010; Kumar et al., 2009; Portmann et al., 2014; Weiss et al., 2008) and ADHD (Chung et al., 2021; Männik et al., 2015; Shiow et al., 2009).

#### 22q11.2

The 22q11.2 locus contains 46 protein coding genes along with other pseudogenes and non-coding areas (Guna et al., 2015). Loss of the 22q11.2 locus has been linked to less cortical gyration. Psychiatric disorders related to attention such as schizophrenia, ASD, ADHD, and generalized anxiety disorder have all been shown to have higher prevalence in those with the 22q11.2 deletion (Schneider et al., 2014). Individuals with 22q11.2 deletion syndrome also seem to have difficulties with RA (Schneider et al., 2014; Simon et al., 2005).

#### APOE

The ε4 haplotype of the *APOE* gene reduces acetylcholine receptor number and possibly diminishes the synthesis of acetylcholine via impaired regulation of phospholipid and/or fatty acid transport (Parasuraman et al., 2002; Poirier, 1996). Middle-aged, non-dementia carriers of ε4 show difficulties with RA tasks (Greenwood et al., 2005).

#### CACNA1C

The *CACNA1C* gene encodes a calcium channel called the CaV1.2 channel. Calcium channels allow selective permeability to calcium ions and thus participate in creating action potentials across cell membranes (Zhang et al., 2023). In the brain, *CACNA1C* affects synaptic plasticity, learning, and memory. Ouyang et al. (2023) describe the connection between *CACNA1C*, schizophrenia, and attention (Meller et al., 2019; Thimm et al., 2011).

#### CHRNA4

This gene encodes a nicotinic acetylcholine receptor that can bind acetylcholine and open an ion-conducting channel across the plasma membrane. The protein can interact with either nAChR beta-2 or nAChR beta-4 (subunits of nicotinic acetylcholine receptors) to form a functional receptor (Winterer et al., 2007). Polymorphisms in this gene are associated with cognitive functions, including attention (Wallis et al., 2009).

#### CNR1

The *CNR1* gene encodes a cannabinoid receptor (CB1) that is expressed in the central and peripheral nervous systems. The receptor helps regulate working memory and attention (Papassotiropoulos and de Quervain, 2011; Ruiz-Contreras et al., 2017; Ruiz-Contreras et al., 2014; Stadelmann et al., 2011).

#### COMT

The G allele at rs4680 (an SNP) produces valine, which is more active in catabolizing—and thus reducing the availability of—dopamine (Axelrod and Tomchick, 1958). *COMT* has been associated with cognitive function generally, including attention (Starr et al., 2007).

#### DRD4

Risk alleles on *DRD4* lead to fewer dopamine receptors via reduced transcription. There is an association between *DRD4* and ADHD (Lowe et al., 2004), and the 7-repeat allele has been associated with RT slopes in RA (Lundwall and Dannemiller, 2015). The *DRD4* receptor has been identified as a potential target for attention-improving medications (Ferré et al., 2022).

#### DTNBP1

Also known as the dystrobrevin-binding protein 1, *DTNBP1* acts by participating in neurotransmitter release, including glutamate. The disruption of neurotransmitter release can result in schizophrenia (Wang et al., 2017). Polymorphisms of *DTNBP1* have been hypothesized to reduce neural activity in prefrontal, temporal, and parietal regions of the brain (Thimm et al., 2010), affecting several cognitive processes, including attention.

#### FMR1

This gene causes fragile X syndrome (Chernenok et al., 2019), which is the most common inherited form of intellectual disability (Hunter et al., 2014; Tassone et al., 2012). Fragile X syndrome occurs when there is an expansion of the CGG trinucleotide within the *FMR1* gene. Such an expansion turns off the *FMR1* genes so that little of the normally resulting protein is produced (Verkerk et al., 1991) and attention is influenced (Cornish et al., 2008; D'Elia et al., 2022; Hunter et al., 2014; Meller et al., 2019; Van der Molen et al., 2012).

#### GRIN2B

This gene encodes a protein found in neurons during prenatal development (Shah et al., 2022). This protein (GluN2B) is essential for creating N-methyl-D-aspartate receptors involved in normal brain development, synaptic plasticity, learning, memory (Ciabarra et al., 1995; Sucher et al., 1995), and attention (Park et al., 2013; Schulz et al., 2012).

#### GRIN3A

Formerly known as *NR3A*, this gene is also involved with N-methyl-D-aspartate receptors. Its involvement in brain development is implied by gene expression peaking in early postnatal life (Das et al., 1998). It is also involved in schizophrenia, which has attentional aspects (Takata et al., 2013).

#### HTT

*HTT* produces the huntingtin protein, implicated in Huntington’s disease. The protein is key in nerve cell signaling, axonal transport, and apoptosis (Nasir et al., 1995; Zhang et al., 2008). *HTT* is essential for normal prenatal brain development and healthy cognition, including attention (Ferré et al., 2022; Linderkamp and Linderkamp-Skoruppa, 2020).

#### MECP2

This gene regulates gene activity by modifying chromatin (Dunican et al., 2007) and thus silencing other genes to help maintain cellular balance (Luikenhuis et al., 2004). Relatedly, *MECP2* is a calcium-dependent transcriptional repressor that alters brain development in ASD (Hawks and Constantino, 2020; Qiu and Cheng, 2010) and related conditions such as Rhett’s syndrome and ADHD (Rose et al., 2019; Yenkoyan et al., 2017).

#### SLC6A3

SLC6A3 controls the number of dopamine transporters, resulting in less dopamine in the synapse and terminating the dopaminergic signal (Giros et al., 1992; Rommelse, 2008). Less dopamine has been associated with greater RA cueing costs for targets in the left hemifield (Bellgrove et al., 2007).

#### ZNF804A

The zinc finger protein 804A gene is expressed throughout the brain during development. Involved in dendritic morphology and synaptic development (Chen et al., 2015; Dong et al., 2021), it has also been studied in relation to attention (Nicodemus et al., 2014).

### Analytic methods

We used Comprehensive Meta Analysis (CMA) software version 4.0 (Borenstein et al., 2022) to analyze our data, using means, standard deviations (*SD*s), sample size, and group membership (e.g., low- versus high-risk) to calculate overall effect size and related statistics. The genetic markers included in the meta-analysis are listed in Table 3. Twenty markers had their means and standard errors estimated from figures and then converted to SDs. We ran comparison analyses for age (child or adult) and neurotransmitter group (dopamine-related or not).

**Table 3:** Genetic markers classified as dopamine-related.

Genetic marker	Source(s)
16p11.2	Portmann et al. (2014)
22q11.2	van Hooijdonk et al. (2022)
COMT	Chen et al. (2004)
DRD4	Nikolova et al. (2011)
DTNBP1	Andreou et al. (2011)
SLC6A3	Reith et al. (2022); Li et al. (2006b)

Determination of dopamine-relatedness relied on National Center for Biotechnology Information at https://www.ncbi.nlm.nih.gov.

## Results

### Description of studies

In the final analysis, 37 studies were included. Data was from 3,228 participants (*M* age = 23.28; age range = 6 months to 70 years^3^). However, the effective sample size in a meta-analysis is the number of studies. We compared 14 studies assessing children (those with participants 0 to 18 years old) to 23 studies assessing adults. We also compared dopamine-related (*n* = 18) to non-dopamine-related (*n* = 19) studies. We did not restrict the meta-analysis to testing any specific gene because we wanted to examine the broader universe of studies to determine an effect size for the influence of genetic evidence on RA generally. See Table 3 for the classification of genetic markers as dopamine-related. There are more non-dopamine related markers in the data from studies on reflexive attention. To keep an approximately equal number of studies per group, we prioritized non-dopamine related genetic markers when selecting markers from already included studies that tested multiple markers. See Table 2.

### Quality of studies included in the meta-analysis

To rate the quality of included studies, we used Q-Genie (Sohani et al., 2015), which is based on standards from genetic experts and journal editors (Freimer and Sabatti, 2003; Li and Meyre, 2013; Little et al., 2009; Nature Genetics Editorial Board, 2005). The developers of the Q-Genie measure (Sohani et al., 2015) used borderline groups regression (Wood et al., 2006) to benchmark the ranges of values that correspond to poor, moderate, and good genetic studies. Note that the questions on Q-Genie can be quite subjective. Sample questions are “Please rate the study on the technical classification of the exposure (i.e., the genetic variant),” “Please rate the study on the non-technical classification of the exposure (i.e., the genetic variant),” and “Please rate the study on description of planned analyses.” Disciplines working with more subjective material are more likely to have lower values accepted as “good” inter-rater reliability.

The complexity of the ratings also influence what value qualifies as the benchmark of “good.” Q-Genie has 11 items, each rated on a 7-point Likert scale. Gwet (2014, p. 171) notes that all benchmarking systems depend on the number of categories into which raters can place the item that is being rated (e.g., coefficients qualifying as good would be higher for two than for seven categories). Sohani and colleagues classify the Q-Genie measure as having “good” reliability (inter-rater reliability as 0.74) and good validity (*ρ* = 0.30, based on correlation between Q-Genie scores and impact factors). We used Q-Genie to judge each study by two reviewers and found that all values for studies were above the cut point for good quality studies.

### Main meta-analysis results

**Figure 3:**
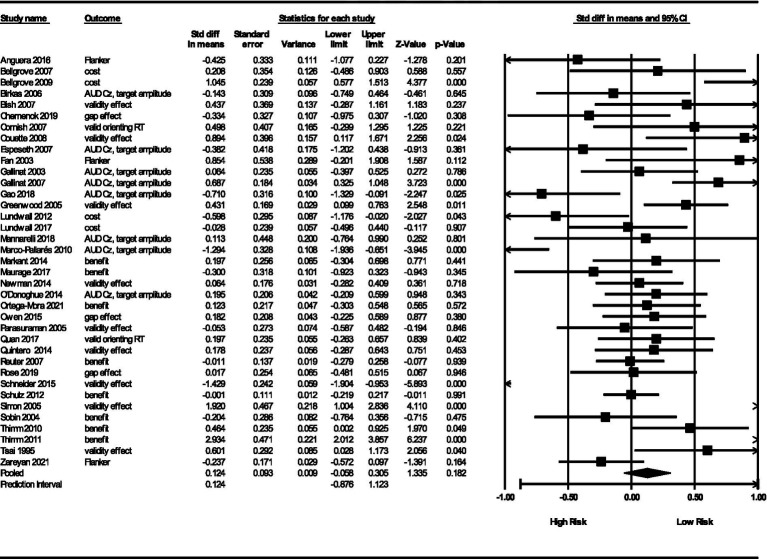
Meta-analytic results and forest plot.

We used a random effects model, which allows us to estimate an overall effect size and potentially generalize to a universe of comparable studies (Hedges and Vevea, 1998; Higgins et al., 2019). This model is also useful in predicting future results of similar studies (Higgins et al., 2009). The analysis produced a point estimate of the mean effect size, *θ* = 0.12 (*SE* = 0.09; 95% *CI* -0.06 to 0.30). This indicates a non-significant overall effect for the influence of genes on RA (*Z* = 1.34, *p* = 0.18). See Figure 3 for the forest plot. Note that the prediction interval crosses zero (−0.97, 1.27). If we assume that the true effects are normally distributed, then we obtain the prediction interval that we can use to anticipate that the true effect of any single new study would fall in this interval (Borenstein, 2020; Higgins et al., 2003; Higgins and Thompson, 2002; IntHout et al., 2016). When the prediction interval varies this way relative to the effect size estimate, the mean effect size becomes less interesting, and we focus instead on what influences the differences between studies. We do this in the section “Subgroup Analyses.”

### Assessment of heterogeneity

Our estimated 𝝉^2^ of 0.23 (*SD* = 0.48) represents the variance in the true effect size and is considered moderate. In addition, the *I*^2^ of 0.80 indicates that 80% of the variance in observed effects reflects variance in true effects rather than sampling error. The Q-value, which tests the null hypothesis that the true effect size is the same in all these studies, is 180.04 (*df* = 36, *p <* 0.001). Using an alpha of 0.10, we reject the null hypothesis that the true effect size is the same in all included studies.

### Publication bias

Publication bias occurs when studies with large effect sizes are more likely to be published, which can cause misleading overall estimates when performing a meta-analysis (Page et al., 2021; Vevea and Woods, 2005) because the data from studies that are well-conducted but non-significant should be included in estimating true effect sizes.

**Figure 4:**
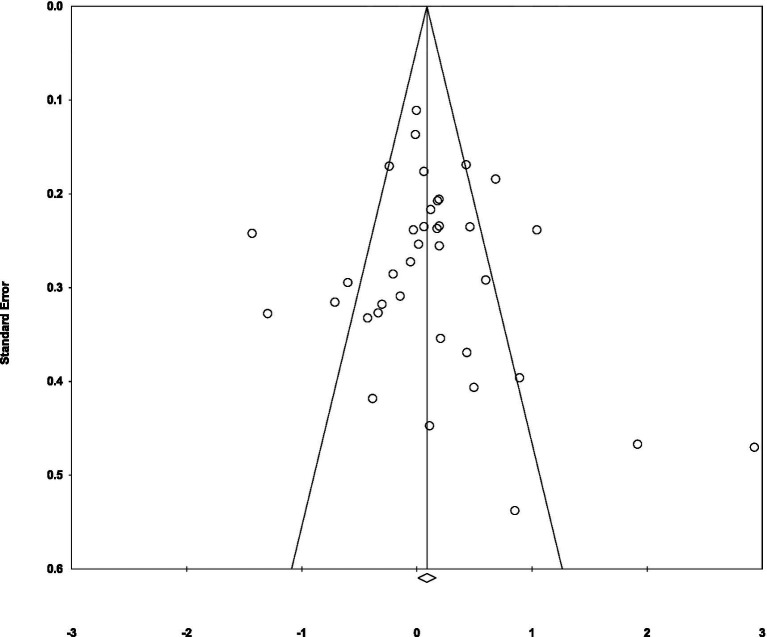
Funnel plot illustrates standard error by Hedge’s g to estimate publication bias.

To investigate the possibility of publication bias, we generated a funnel plot to assess left–right asymmetry and found asymmetry (see Figure 4). We also ran a Begg and Mazumdar’s rank correlation test, which quantifies publication bias by looking for an inverse correlation between study size and effect size (Begg and Mazumdar, 1994; Boccia et al., 2010). We found no correlation (using continuity correction; Kendall’s 𝝉_b_ correlation = 0.02, *p* = 0.86).

### Sensitivity analyses

To better understand our results, we explored sensitivity to outlier studies, we also used one-study-removed methodology. Sensitivity analyses simply indicate how much results would change when studies are removed one at a time (Vevea and Woods, 2005). However, no matter which study we removed, the overall effect remained essentially the same (estimated effect sizes [*θ*s] ranged from 0.07 to 0.16, *p*-values ranged from 0.05 to 0.42). This suggests that the non-significance of our meta-analysis is not driven by the inclusion of any one study. Although there is no overall effect for genes on RA, we wanted to explore the possibility of effects dependent on the type of outcome used, age group (child or adult studies), and neurotransmitter groups (genes affecting dopamine or non-dopamine-related neurotransmitters).

### Subgroup analyses

Group comparisons were performed based on the interests of study authors. In addition to comparison by outcome type, we were interested in age (child or adult) and in the neurotransmitter dopamine, including genetic studies that point to the importance of dopamine in RA (Bellgrove et al., 2007; Bellgrove et al., 2008).

#### Outcome type

**Figure 5:**
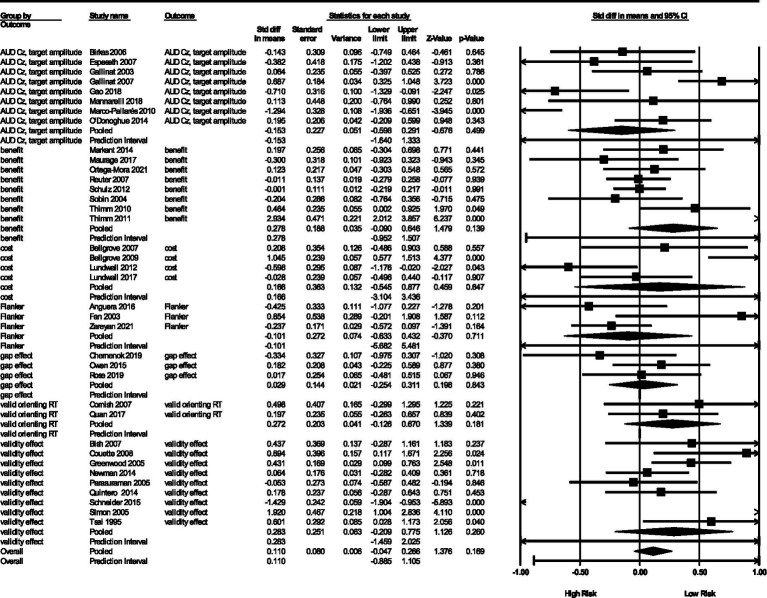
Forest plot by outcome type.

There were seven types of outcomes. Each have their own point estimates, prediction intervals, and heterogeneity statistics. This can be seen in the pooled results at the bottom of each outcome type in Figure 5. Here we review some interesting findings.

First, the significance of genetic studies in predicting RA did not vary by outcome type. The most significant outcome was valid RT with a *θ* = 0.27 (*SE* = 0.20, 95% *CI* = 0.13, 0.67) and a *p*-value of 0.04. It has a non-calculatable prediction interval, probably due to a low number of studies included (*n* = 2). Gap effect had a similar problem (*n* = 3 studies).

Second, associated heterogeneity analyses indicate that costs and validity effects have the highest between-study variance, 𝝉^2^ = 0.45, SD = 0.67 and 𝝉^2^ = 0.48, *SD*s = 0.69, respectively. The outcome types with low between-study variance was flanker congruency effect (𝝉^2^ = 0.12, *SD* = 0.34), excepting gap effect and valid RT which did not have calculable between-study variance. We also obtained *I*^2^ statistics (81–86% for the first three outcomes and 54% for flanker congruency effects), but note that they do not represent variance in the studies, but the proportion of variance in true effects relative to variance in observed effects.

#### Age group

**Figure 6:**
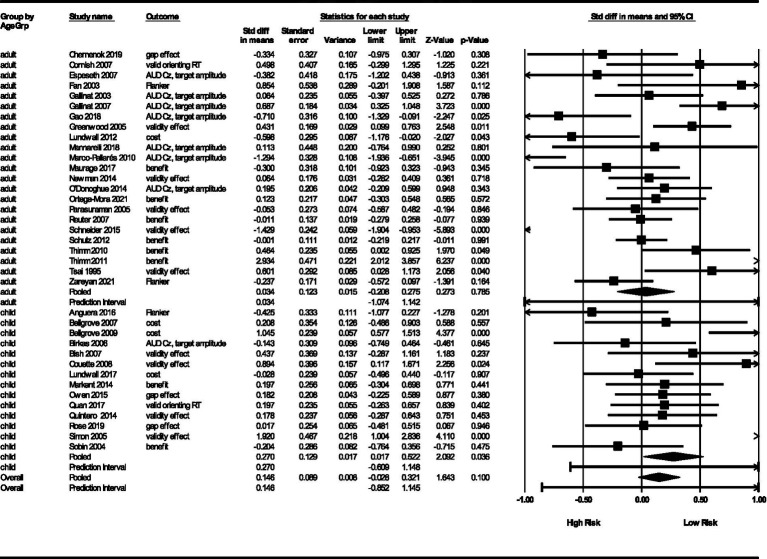
Results by age group.

For children, we obtained a point estimate of *θ* = 0.27 (*SE* = 0.13, 95% *CI* = 0.02, 0.52). This is significant (*Z* = 2.09, *p* = 0.04) but has a wide prediction interval (−0.61, 1.15). Alternatively, for adults, we obtained a point estimate of *θ* = 0.03 (*SE* = 0.12, 95% *CI* = −0.21, 0.28). This was not significant (*Z* = 0.27, *p* = 0.78) and also has a wide prediction interval. See Figure 6. The different effects on children and adults are intriguing and addressed in the Discussion section.

In addition, variances differed across age groups with adult studies showing more variance (𝝉^2^ = 0.27, *SD* = 0.52) than child studies (𝝉^2^ = 0.15, *SD* = 0.38). We also obtained *I*^2^ heterogeneity statistics (84% for adult studies and 65% for child studies). These represent the proportion of variance in true effects relative to variance in observed effects. The prediction intervals for adults (−1.07 to 1.14) and children (−0.61, 1.15) both indicate that future studies are likely to have an effect size that would fall in this interval (Borenstein, 2020; Higgins et al., 2003; Higgins and Thompson, 2002; IntHout et al., 2016). So, while the significant child studies are intriguing, there is not yet assurance that future studies would also be significant.

#### Dopamine-related genetic markers

**Figure 7:**
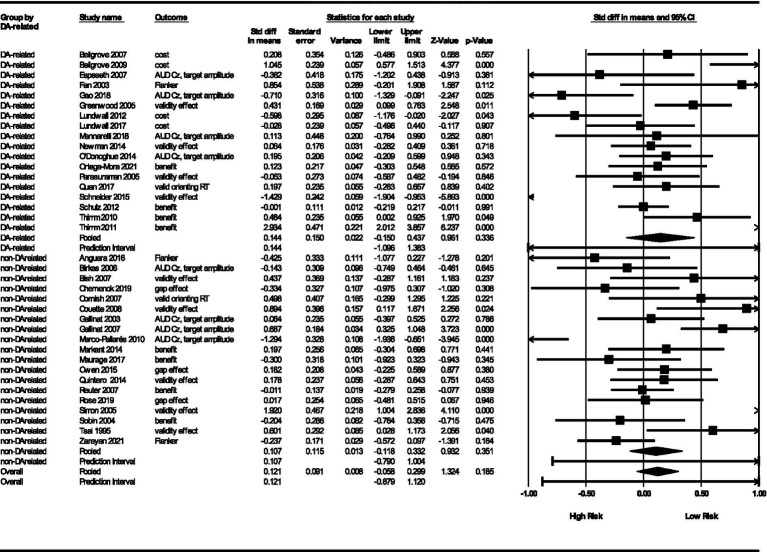
Results by dopamine-related group.

Lastly, we were curious if genetic markers related to the availability of dopamine had a lower or higher effect than non-dopamine genetic markers. For non-dopamine related genetic markers, we obtained a point estimate of *θ* = 0.11 (*SE* = 0.12, 95% CI = −0.12, 0.33). This is not significant (*Z* = 0.93, *p* = 0.35) and has a prediction interval from −0.79, 1.00. For dopamine-related genetic markers, we obtained a point estimate of *θ* = 0.14 (*SE* = 0.15, 95% CI = −0.15, 0.44), which is similarly not significant (*Z* = 0.96, *p* = 0.34) and has a prediction interval (−1.10, 1.38). Figure 7 illustrates the results. As with age group, variances differed across age groups with dopamine-related studies showing more variance (𝝉^2^ = 0.32, *SD* = 0.56) than non-dopamine studies (𝝉^2^ = 0.17, *SD* = 0.41). *I*^2^ heterogeneity statistics did not appear to vary substantially (85% for dopamine-related studies and 72% for non-dopamine-related studies). The prediction intervals for dopamine-related studies (−1.10 to 1.38) and non-dopamine-related studies (−0.79, 1.00) both indicate that future studies are likely to have an effect size that would fall in this interval (Borenstein, 2020; Higgins et al., 2003; Higgins and Thompson, 2002; IntHout et al., 2016).

## Discussion

Our study is the first to summarize the literature concerning possible genetic influence on RA. Although we did not find a significant overall effect for the influence of genes on RA, we explored subgroups which did show a significant differences. We address possible reasons for the whole-group non-significant difference in light of subgroup findings. This includes examining differences by outcome type and if moderators such as age and the influence of dopamine played a role in estimates of effect size. In total, we synthesized evidence from articles from 1994 to 2023 that studied RA in human participants and that analyzed the influence of at least one genetic marker.

Although the overall meta-analysis did not show a statistically significant effect size for a general genetic influence on RA, children did show a genetic effect on RA while adults did not. This difference is interesting in itself, and we discuss it in more depth below. As noted earlier, when results vary like this, the mean effect size of combined groups becomes less interesting, and we focus instead on what influences the differences between studies. Here, we examine the results by comparing genetic influence on RA depending on outcome type, age group, and the dopamine-relatedness of the gene (Godfrey et al., 2018; Lehmann et al., 2022; Li et al., 2006a; Little et al., 2012).

### Group analyses

#### Outcome type

The overall heterogeneity in outcome types in our meta-analysis was moderate. However, several outcomes had high within-outcome heterogeneity (such AUD Cz, costs, and validity effect). While this might mean that these measures do not measure the same construct across studies, it could also mean that the studies vary for other reasons, such as sampling from different populations or assessing different genes. The remaining outcome measures had low or moderate within-outcome heterogeneity. Overall, out meta-analysis offers some support for the idea that the different ways of measuring RA are related, as theory implies (Lyytinen et al., 2002; Posner, 1980; Sokolov, 1963, 1966; Voronin and Sokolov, 1960). Since we found no other studies that tested this idea, this may be an important contribution.

Related is the question, “which measures were more often successful at detecting differences in RA?” Examining Figure 5 again, we see that outcomes with pooled effects in the opposite direction from expected include AUD Cz target amplitude and flanker congruency effect. Some outcomes also had a smaller proportion of studies producing significant effects (i.e., flanker, gap effect, and valid RT). Only validity effect had more than half its studies producing significant results, and one was in the unexpected direction. Overall, validity effect seems to be slightly better at detecting the influence of genes on RA, although its pooled effect size is not significant.

#### Age group

Our most interesting finding is that estimates of effect size differed by age group. Children had effect sizes significantly different from zero while adults did not. This suggests that studies of genetic influence on RA is children is more apparent in children. It is likely that the same genotype has different influences on phenotype across development (Markant et al., 2014; Tang et al., 2020). For example, we know that RA develops in tandem with cholinergic and dopaminergic polymorphisms expressed during early life (Burstein et al., 2021; Markant et al., 2014; McKinnon and Nathanson, 1995). Had the same genotypes been tested at multiple ages, ideally in a longitudinal study, it would be possible to determine if the same genotype exerts a stronger influence on RA at different points in the lifespan.

We were not able to find a similar effect size for genetic influence on adult RA. One possibility is that there were four studies with considerable effects in the opposite direction of the expected effect in the adult age group. Two were AUD Cz studies (Gao et al., 2018; Marco-Pallarés et al., 2010). Since AUD Cz was likely to be an outcome in child studies, this may represent a confound. Another possibility is that a genotype thought to be an advantage at one age is not an advantage at another age. One illustration of this general concept is that some studies suggest that the APOE ε4 variant, typically associated with Alzheimer’s disease, may confer cognitive advantages in younger individuals (Oriá et al., 2010; Wright et al., 2003; van Exel et al., 2017).

**Figure 8:**
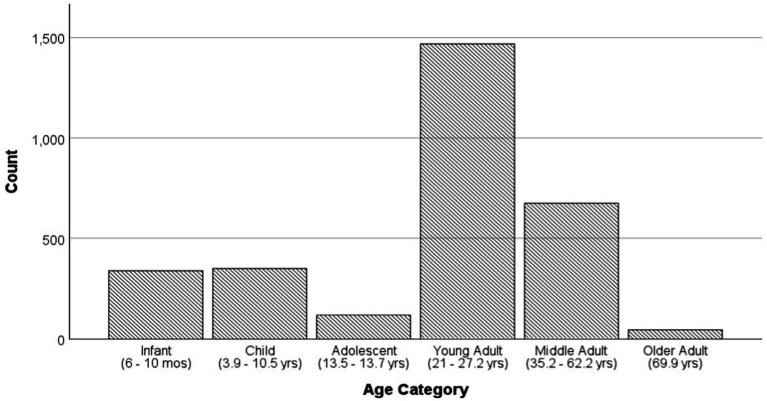
Age distribution by study sample sizes. This is a distribution of the average age per study (individual ages were not provided).

Likely due to understandable difficulty in using the same measures for young infants as for older children and adults, we were not able to find any genetic studies of RA in infants younger than 6-months-old (see Figure 8). This is less than ideal because birth to 6-months-old is when RA might show the most development (Atkinson and Braccick, 2012; Colaizzi et al., 2014; Courage et al., 2006; Kannass et al., 2006; Posner et al., 2012; Richards, 2005; Varga et al., 2010). If infants had been included, we might have seen stronger effects of age on RA and been able to determine if they are linear.

#### Dopamine-related genetic markers

We found no evidence that dopamine-related or non-dopamine-related genes impacted RA. The pooled effect sizes for both groups are similar prediction intervals for both were wide, suggesting future studies might achieve results anywhere in a relatively large band that spans zero. Many researchers study the effects of genetic polymorphisms on human behavior because the markers influence the availability of neurotransmitters. Dopamine, in particular, is a common biological pathway to behavior named in genetic studies (Blum et al., 2000). In this meta-analysis, dopamine-related genes showed more variance than non-dopamine-related genes. Several studies suggest non-dopamine-related genes also have significant influence on RA. For example, norepinephrine is crucial for maintaining alertness, arousal, and attention and that individuals with ADHD may have lower norepinephrine levels, contributing to difficulties with sustained attention (Beane and Marrocco, 2004). Similarly, acetylcholine plays a significant role in attentional arousal (Beane and Marrocco, 2004; Oberlin et al., 2005). Serotonin also influences attention (Oades and Müller, 1997). Glutamate is an excitatory neurotransmitter involved in various brain functions such as attention (Cid-Jofré et al., 2022; Park et al., 2013). With more studies of genetic influence on RA, researchers may be able to examine the effects of these neurotransmitters individually.

However, fewer studies are published associating or attempting to associate noradrenergic, cholinergic, serotoninergic, and glutamatergic genes to RA. A general university database search for studies on genes predicting RA found acetylcholine as *n* = 160 and norepinephrine as *n* = 180 compared to dopamine (*n* = 483). This may be confirmation bias in the sense that there is a tendency to investigate neurotransmitters which have already been found as significant by other researchers (Bishop, 2020; Radua and Fullana, 2022). Dopamine’s role is particularly well-studied in conditions like ADHD.

### Limitations

Correct interpretation of meta-analyses is important to avoid invalid generalizations (Aikins et al., 2001; Ramasamy et al., 2008). The biggest caveat to keep in mind is that we can only generalize our results to studies comparable to those in our analysis. This includes studies with the same mix of samples, outcomes, and predictors, which can be complicated (van Dijk et al., 2024; Wang et al., 2020). In this case, our analysis is complicated by the fact that it cannot point to any specific genetic markers as more important than others. Instead, we considered the biologic effect of each marker (Collins et al., 2003; Otero-Carrasco et al., 2024) by looking at its influence on the availability of dopamine.

Another limitation was the inability to of our team to comprehend all relevant languages. The lack of non-English studies is an issue because it suggests the likelihood that genetic and cultural groups may be missing form inclusion. Including them would have allowed us to access more diverse studies and improve our estimates.

Although all included studies had reasonable quality ratings using the Q-Genie measure (Sohani et al., 2015), 6 of 37 studies were considered under-powered by two raters using the Q-Genie measure. This relates to a single item on Q-Genie and thus did not strongly impact study quality ratings.

Finally, there were concerning aspects of the studies that were not assessed by Q-Genie. For example, 25 of 37 studies did not report ethnicity and none reported socioeconomic status or income, which may sometimes be associated with impaired attention. Six of the studies included some aspect of age besides mean and standard deviation (e.g., including only the standard deviation). Relatedly, we were not able to find any studies with a sample size larger than 336. Given that 21 of the 37 studies were published prior to 2013, which is arguably when the replication crisis became common knowledge (Asendorpf et al., 2013), larger studies reporting on more details would likely be helpful. Future genetic meta-analyses may need alternative means of rating studies that consider factors important to avoiding failure to replicate.

### Implications

Our main finding emphasizes that the influence of genes on RA varies by age group. Additionally, some outcome measures show more heterogeneity than others and this should be considered by researchers when choosing outcomes. Similarly, studies testing non dopamine-related genes had lower heterogeneity than those testing dopamine-related genes. For future candidate gene studies, we recommend including genes associated with a wider variety of neurotransmitters. Our strongest recommendation is that more complete reporting is needed within studies, including age and ethnicity. We also urge more effort be placed on recruiting and reporting diverse ethnic groups. When conducting a meta-analysis, we advise collaborating with colleagues who understand diverse languages (e.g., Chinese) at the level of academic journal writing. While our findings are interesting, it remains important to study specific neurotransmitters rather than neurotransmitters at a binary level.

## Conclusion

In summary, we combined past RA gene association studies to consolidate what we can learn from them. Our method of analysis allowed for better estimates of effect sizes than individual studies. The significant effect sizes we found for children as opposed to adults in how well genes predict RA outcomes could benefit future studies in the development of this foundational cognitive ability. We believe these results are important, in part because RA is foundational to development and higher cognitive processes.

## Data Availability

Publicly available datasets were analyzed in this study. Data were extracted from published articles.

## Footnotes

^1^Note that RA has sometimes been called “orienting” or “selective attention” in the literature; however, these terms also refer to voluntary orienting and selection. While we examined studies referring to orienting and selective attention to determine if they included examination of RA, RA is our preferred term.

^2^This was because we had no colleague willing and able to search for, comprehend, and extract effect sizes from non-English languages.

^3^Because not all studies provide age ranges, we describe the most commonly reported parameter, which was mean age.
